# Healthcare-Associated Stenotrophomonas maltophilia Bacteraemia: Retrospective Evaluation of Treatment and Outcome

**DOI:** 10.7759/cureus.18916

**Published:** 2021-10-20

**Authors:** Tekin Tuncel, Halis Akalın, Melda Payaslıoğlu, Emel Yılmaz, Esra Kazak, Yasemin Heper, Cüneyt Özakın

**Affiliations:** 1 Infectious Diseases and Clinical Microbiology, Bursa Uludağ Üniversitesi, Bursa, TUR; 2 Medical Microbiology, Bursa Uludağ Üniversitesi, Bursa, TUR

**Keywords:** sepsis, prognostic risk factors, bacteraemia, bloodstream infections, s. maltophilia

## Abstract

Introduction

*Stenotrophomonas maltophilia *(SM) is one of the common gram-negative pathogens that cause nosocomial infections. The aim of the present study is to evaluate the treatment and outcome of SM bacteraemia.

Materials and Methods

We retrospectively evaluated antimicrobial treatment in adult patients with nosocomial SM bacteraemia, with the 14th and 30th-day mortality as the outcome.

Results

In total, 140 adult patients with SM bacteraemia who were diagnosed between January 1, 2002, and December 31, 2016 were enrolled in the present study. Seventy-one (50.7%) patients were in the intensive care unit (ICU). The 14th and the 30th-day mortality rates were 32.9% (n=46) and 45.7% (n=64), respectively. Female sex (OR, 7.47; 95% CI 1.61-34.47, p<0.01), steroid use within the last month (OR, 10.2; 95% CI 1.27-82.27, p=0.029), Pittsburgh bacteraemia score (PBS) ≥4 (OR, 39.9; 95% CI 4.96-321.32, p<0.001) and solid organ malignancy (OR, 9.6; 95% CI 1.73-53.72, p<0.01) were independent risk factors for 14th day mortality. Removal of the catheter was an independent protective factor for both 14th (OR, 0.05; 95% CI 0.22-0.010, p<0.001) and 30th day (OR, 0.039;95% CI 0.164-0.009, p<0.001) mortality. We did not detect any difference between treatment regimens including trimethoprim-sulfamethoxazole (TMP/SMX) or levofloxacin in terms of mortality. We found that TMP/SMX and levofloxacin combination did not significantly improve patient prognosis.

Conclusion

Due to the high mortality rates associated with nosocomial SM bacteraemia, adequate antibiotic therapy should be initiated immediately in the suspicion of infection, and prompt removal of any indwelling central venous catheter is important.

## Introduction

*Stenotrophomonas* species can survive on humans, animals and plants, and they may be isolated from soil and water [1]. These bacteria may also exist on different surfaces in hospital environments and can cause nosocomial infections [2,3]. Recent studies report that *Stenotrophomonas maltophilia* (SM) causes bacteraemia and pneumonia, and whose presence has been gradually increasing over the last decade in hospitalized and critically ill patients [4]. *Stenotrophomonas maltophilia *has intrinsic resistance against beta-lactams, carbapenems and aminoglycosides, which are frequently used for empirical treatment of nosocomial infections. Previous studies indicate that mortality rates vary between 21% and 69% for cases with SM bloodstream infections [5].

Previous studies show that higher acute physiology and chronic health evaluation II (APACHE II) scores, higher sequential organ failure assessment (SOFA) scores, thrombocytopaenia, use of carbapenem within the last three months, septic shock, admission to intensive care unit (ICU), neutropenia, polymicrobial bacteraemia with *Enterococcus spp. *and inadequate empirical antimicrobial treatment are responsible for poor prognosis, whereas removal of the central venous catheter affects prognosis positively [6-12]. Since different outcomes were measured in studies of prognostic risk factors and effects of adequate treatment on prognosis, further studies should be performed on the treatment of SM infections and prognostic risk factors.

The aim of the present study is to retrospectively evaluate the treatment and outcome of nosocomial SM bacteraemia.

## Materials and methods

The present study was approved by the Ethics Committee for Clinical Researches of Bursa Uludağ Üniversitesi Medical Faculty (No.:2016-19/8) on November 28, 2016. After approval, patient information was screened retrospectively from the electronic filing system of our hospital.

We included adult patients (>18 years of age) with at least one strain SM isolation in the blood culture and who met at least two systemic inflammatory response syndrome (SIRS) criteria. Only a patient’s first bacteraemia episode was enrolled into the study; further episodes were excluded [13].

Sepsis or septic shock was differentiated according to the sepsis-1 criterion for all patients [14]. The use of prednisone at 20 mg/day for two weeks or 30 mg/day for one week within the last month was considered a risk factor. Patients with a neutrophil count below 500/mm³ were considered neutropenic [11]. Antibiotic use for at least 48 hours within 14 days of the diagnosis was considered antibiotic use. The use of carbapenem within the last three months was also evaluated.

In the presence of active infection, the focus was accepted as a source of bacteraemia according to SM isolation just before a positive blood culture or on the same day as a positive blood culture [11]. SM isolation in the blood culture from peripheral blood and catheter or in semiquantitative cultures of the catheter tip (Maki’s semiquantitative culture method) was accepted as catheter-related bacteraemia in the absence of any other infection focus [15,16]. Nosocomial bacteraemia was defined as the appearance of infection at least 48 hours after admission and the absence of infection at admission [8]. The cut-off value for deep endotracheal aspirate was ≥100000 colony-forming unit (CFU)/mL.

The use of ineffective (bacteria were considered resistant or intermediate in antibiotic susceptibility tests) antibiotics within the first 24 hours after collection of a blood sample for culturing was considered as inadequate treatment; use of effective antibiotics (bacteria were considered susceptible in antibiotic susceptibility tests) was considered as adequate treatment. The presence of at least one effective antibiotic in combination treatments was accepted as an adequate treatment [17]. If an adequate treatment was started between 24 to 48 hours after collection of blood sample for culturing, it was defined as late adequate treatment. And if adequate treatment was started after 48 hours after collection of blood sample for culturing, it was defined as very late adequate treatment. The outcome of the study was mortality at the 14th and 30th day after blood sample was collected for culturing. The dosage of trimethoprim/sulfamethoxazole (TMP/SMX) was calculated in terms of 15 mg/kg TMP per day.

The Charlson comorbidity index was used to detect the grade of underlying disease for patients in the inpatient clinic and APACHE II score (at the time of admission to ICU) was used to evaluate the physiological state and chronic diseases for the patients in the ICU. The Pittsburgh bacteraemia score (PBS) was calculated on the day of collecting a blood sample for culturing [18].

At least two bottles were collected per patient, and blood samples were incubated for five days. Blood samples were placed in BACTEC 9000 (Becton Dickinson, INC, Sparks, MD, USA) blood culture vials. Deep tracheal aspirate (DTA) cultures and positive blood cultures were seeded in sheep blood agar and eosin-methylene blue agar. The DTA cultures were quantitatively evaluated, and the threshold value was 100,000 colony forming unit (CFU)/ml. The Phoenix Expert System (Becton Dickinson, INC, Sparks, MD, USA) was used to identify and determine antibiotic susceptibility. Antibiotic susceptibility tests were evaluated according to the Clinical and Laboratory Standards Institute (CLSI) criteria until 2014, and they were evaluated according to the European Committee on Antimicrobial Susceptibility Testing (EUCAST) after 2014.

Statistical analysis

Compliance of the variables to normal distribution was evaluated by Shapiro-Wilk tests. Continuous variables were expressed as mean ± standard deviation or median (minimum-maximum). Mann-Whitney U tests or t-tests were used to compare continuous variables between groups. Categoric variables were expressed in frequency and percent; Pearson's chi-square test and Fisher's exact chi-square test were used for comparisons between groups. Binary logistic regression analysis was performed to determine the independent risk factors for 14th and 30th-day mortality. The variables were included in a logistic regression model forwardly to determine risk factors. The variables that were found to be significant were considered independent risk factors. The logistic regression model was significant (p<0.001). Statistical analyses were carried out by using SPSS (IBM Corp. Released 2012. IBM SPSS Statistics for Windows, Version 21.0. Armonk, NY: IBM Corp.) and values of p<0.05 were considered statistically significant.

## Results

In total, 172 adult patients (>18 years of age) with at least one SM isolation from blood cultures were identified in our hospital between January 1, 2002 and December 31, 2016. As the files of 15 patients were not accessible, 10 patients did not meet at least two systemic inflammatory response syndrome (SIRS) criteria along with positive blood culture and seven patients died on the day of diagnosis, 140 patients were included in the study. Seventy-one (50.7%) patients were in the ICU and 49.3% of the patients were in the inpatient clinic. The demographic and clinical characteristics of the patients are presented in Table 1.

**Table 1:** Demographic and clinical characteristics of the patients *SM isolation from any clinical sample of the patient except blood culture within the last one month. MV: Mechanical ventilation, TPN: Total parenteral nutrition, APACHE II: Acute physiological and chronic health evaluation, CVC: Central venous catheter, PBS: Pittsburgh bacteraemia score, SM: *Stenotrophomonas maltophilia *

Demographic characteristics	Number of patients (n)	Percentage (%)
Age (median, min.-max.)	54 (18-84)	
Gender: Female & Male	59 & 81	42.1 & 57.9
MV	71	50.7
CVC	101	72.1
TPN	58	41.4
Urinary catheter	103	73.6
Nasogastric catheter	68	48.6
Drainage catheter	54	38.6
History of surgery within last month	43	30.7
Sepsis	93	66.4
Septic shock	47	33.6
PBS ≥ 4	59	42.1
PBS< 4	70	50
APACHE II ≥20	25	17.9
APACHE II <20	46	32.9
Antibiotic use within last 14 days	127	90.7
Use of carbapenem within last three months	96	68.6

Various underlying diseases were detected in 115 patients (82.1%). The most common underlying diseases were solid organ malignancy (42 patients; 30%), haematological malignancy (32 patients; 22.9%), and cerebrovascular disease (24 patients; 17.1%). Multiple underlying diseases were detected in 43 patients (30.7%).

The most common source of SM infection in the patients was catheter-related bacteraemia (CRB), which occurred in 29 patients (20.7%), followed by pneumonia in nine patients (6.5%) and intraabdominal infection in eight patients (5.7%). Among 94 patients (67.1%) with an undetected source, 66 (47.1%) had a central venous catheter for more than 48 hours.

In 93 patients (66.4%), only SM was isolated, indicating monomicrobial bacteraemia, whereas other microorganisms were isolated along with SM in 47 patients (33.6%). The most common bacteria isolated along with SM were coagulase-negative *Staphylococcus spp.* (CNS) in 26 samples of 23 patients (16.4%) with two different CNS isolated from three patients). The second most common bacteria isolated with SM were *Enterococcus spp.* in 10 patients (7.1%).

The antibiotic susceptibility test results of SM strains isolated from patient blood are presented in Table 2. Colistin resistance was not detected.

**Table 2:** Antibiotic susceptibility test results of S. maltophilia strains isolated from the blood

Antibiotic	Sensitive n (%)	Total (n)
Trimethoprim-sulfamethoxazole (TMP/SMX)	133 (95)	140
Levofloxacin (LEV)	88 (91.6)	96
Ciprofloxacin	13 (28.2)	46
Ceftazidime	27 (42.8)	63
Cefoperazone-sulbactam	12 (38.7)	31
Cefepime	7 (15.5)	45
Ticarcilline clavulanate	9 (18)	50
Piperacillin-tazobactam	8 (16.6)	48
Chloramphenicol	13 (65)	20

Empirical antibiotic treatment was started in 111 patients. Adequate empirical treatment was started in 32 patients (22.9%); inadequate empirical treatment was started in 79 patients (56.4%). Of the patients treated empirically, monotherapy was preferred in 59 patients (42.1%), whereas combination therapy was preferred for 52 (37.1%).

Empirical treatment was not started and current antibiotic treatment was continued for 29 patients (20.7%). Of these patients, three received late adequate antimicrobial treatment, and 26 received very late adequate antimicrobial treatment.

Of the 32 patients who received adequate empirical treatment, monotherapy and combination therapies were preferred for nine and 23 patients, respectively. Colistin was included in the therapy of 14 patients who received adequate empirical treatment, including three patients who received monotherapy and 11 patients who received combination therapy. Trimethoprim/sulfamethoxazole was started empirically in 11 patients, including three patients who received monotherapy and eight patients who received combination therapy.

Fifty-four of 79 patients who received inadequate empirical treatment received late or very late adequate treatment, whereas 25 patients received neither late nor very late adequate treatment during follow-up; 20 (80%) of these patients died within 30 days.

With the exemption of the 25 patients who did not receive any adequate treatment, adequate treatment regimens and mortality rates for the remaining 115 patients (32 patients who started adequate empirical treatment and 83 patients who did not receive any empirical treatment or received late or very late adequate treatments following inadequate empirical treatment) are presented in Table 3.

**Table 3:** Treatment regimens and mortality rates in patients who received adequate treatment a,d: p=0.2, p=0.5; a,b: p=0.6, p=0.6; a,c: p=0.7, p=0.1; b,c: p=1,p=0.32; b,d: p=0.4,p=0.3  c,d: p=0.6, p=0.1 (p values are provided for 14th and 30th-day mortality) TMP/SMX: Trimethoprim/sulfamethoxazole, LEV: Levofloxacin

Treatment	Number of patients	14th-day mortality n (%)	30th-day mortality n (%)
TMP/SMX + LEVᵅ	38	7 (18.4)	16 (42.1)
Regimens including TMP/SMX (except LEV)ᵇ	49	11 (22.4)	18 (36.7)
Regimens including LEV (except TMP/SMX) c	17	4 (23.5)	4 (23.5)
Other adequate treatment regimens without TMP/SMX or LEVᵈ	11	4 (36.3)	6 (54.5)
Total	115	26 (22.6)	44 (38.2)

Mortality rates for the 14th and 30th day were 32.9% (n=46) and 45.7% (n=64), respectively. The highest 14th and 30th-day mortality rates were detected in patients with pulmonary infections (44.4%, 55.6%) and patients with intraabdominal infections (25%, 62.5%) as well as the group with an undetected bacteraemia source (36.2%, 45.7%); the lowest mortality rate was detected in the patients with CRB (20.7%, 37.9%).

When polymicrobial infections were excluded, there was no significant effect of adequate empirical treatment on 14th and 30th-day mortality rates for patients with monomicrobial bacteraemia (p=0.812 and p=0.944, respectively).

There was no significant difference between the patients (n=32) for whom adequate empirical treatment was started and the patients (n=79) for whom inadequate empirical treatment was started in terms of both 14th and 30th-day mortality rates, except the group (n=29) who did not receive any empirical treatment (p=0.838 and p=0.952, respectively).

Comparison of the patients (n=25) who did not receive any adequate treatment and the patients who received adequate treatment revealed that 14th and 30th-day mortality rates were significantly lower in the groups receiving adequate treatment (Table 4).

**Table 4:** Comparison of patients who did not receive any adequate treatment and those who received adequate treatment in terms of 14th and 30th-day mortality rates *Each group was compared with the patient group who "did not receive any adequate treatment". **For 14th-day mortality ***For 30th-day mortality

Patient groups	14th-day mortality n (%)	30th-day mortality n (%)	P-value*
Patients who did not receive any adequate treatment (n=25)	20 (80)	20 (80)	
Those whose adequate empirical treatment was started (n=32)	12 (37.5)	16 (50)	0.001** 0.02***
Patients on whom inadequate empirical treatment was started and received adequate treatment during follow-up (n=54)	8 (14.8)	20 (37)	<0.001** <0.001***
Patients on whom inadequate empirical treatment was started or those who did not have any empirical treatment and received adequate treatment during follow-up (n=83)	14 (16.9)	28 (33.7)	<0.001** <0.001***
Patients who received adequate treatment (n=115)	26 (22.6)	44 (38.3)	<0.001** <0.001***

To detect risk factors for mortality, we compared the patients who survived or died. The risk factors for 14th-day mortality were detected as being ≥65 years of age, of the female sex, PBS ≥4, use of steroids within the last month, polymicrobial infection, septic shock, and solid organ malignancy in terms of 14th-day mortality (p=0.029, p=0.041, p<0.001, p=0.002, p=0.012, p<0.001, p=0.005 respectively). Removal of the central venous catheter was a significant indicator of a good prognosis (p<0.001).

The risk factors for mortality were detected as age ≥65 years, urinary catheter, PBS ≥4, septic shock, and solid organ malignancy in terms of 30th-day mortality (p=0.049, p=0.008, p<0.001, p<0.001, p=0.012, respectively). Removal of the central venous catheter was an indicator of a good prognosis (p<0.001).

Logistic regression analysis for 14th-day mortality revealed female sex, PBS ≥4, steroid use within the last month, and solid organ malignancy as independent risk factors, whereas removal of the central venous catheter was detected as an independent protective factor (Table 5).

**Table 5:** Independent risk factors in S. maltophilia bacteraemia for 14th-day mortality PBS: Pittsburgh bacteraemia score, CVC: Central venous catheter, CI: Confidence interval

Risk factor	OR (95 % Cl)	P-value
Female gender	7.47 (1.61-34.47)	0.01
PBS ≥ 4	39.9 (4.96-321.32)	0.001
Use of steroids within the last one month	10.2 (1.27-82.27)	0.029
Solid organ malignancy	9.6 (1.73-53.72)	0.01
CVC removal	0.05 (0.22-0.010)	<0.001

PBS ≥4 (OR, 10.9; 95 % CI 2.88-41.43, p<0.01) was detected as an independent risk factor for 30th-day mortality, whereas removal of the central venous catheter was an independent protective factor (OR, 0.039; 95 % CI 0.164-0.009, p<0.001).

34.8% of patients admitted to the inpatient clinic had died by the 14th day. The logistic regression analysis for 69 patients admitted to the inpatient clinic demonstrated that use of steroids within the last month (71.9 [1.36-3788], p=0.034) and high Charlson's score (2.03 [1.09-3.76], p=0.024) were independent risk factors for 14th-day mortality.

Of patients admitted to the ICU, the 14th-day mortality rate was 31% (n=22). The presence of solid organ malignancy was an independent risk factor for 14th-day mortality (OR, 11.4; 95% CI 1.57-83.3, p=0.016). Central venous catheter (CVC) removal was an independent protective factor (OR, 0.02; 95% CI 0.14-0.003, p<0.001).

## Discussion

Previously reported mortality rates for patients with bloodstream infections due to SM varies between 21% and 69% [5, 19]. For patients with haematological malignancy, the mortality rate has been reported as more than 60% [20]. In the present study, the 14th and 30th-day mortality rates were 32.9% and 45.7%, respectively, which is consistent with the literature. The wide range of mortality rates may depend on heterogeneity of the patient groups (ICU vs. inpatient clinic, type of ICU, haematological malignancy, solid organ malignancy, transplantation patients, mixed populations) and differences in study designs.

The present study and previous studies demonstrated that the use of antibiotics (including carbapenems) and CVC is high in patients with SM bacteraemia [6-10,21,22].

*Stenotrophomonas maltophilia* is often the cause of infection in patients with solid organ malignancy, haematological malignancy, or neutropenia as well as patients who have received immunosuppressive therapy [10,23]. We detected solid organ malignancy in 30% of patients and haematological malignancy in 22.9% of patients in this study.

Empirical treatment was started in 111 patients, and this treatment was adequate in 32 patients (22.9%). Wang et al. [9] reported adequate empirical treatment rates as 8.1%, whereas Jeon et al. [10] and Hotta et al. [6] reported these rates as 9.9% and 31.5%, respectively. The cause for lower rates might have been due to the antibiotic resistance pattern.

In the present study, we demonstrated that adequate empirical treatment does not lead to a statistically significant difference in mortality when compared to inadequate treatment. Some studies suggest that the severity of underlying diseases of patients before and during bacteraemia affects mortality [6,8,10,24]. Only Sumida et al. [7] reported inadequate treatment as an independent risk factor for mortality.

In a study of 142 patients, Jeon et al. [10] detected higher SOFA scores as a 28th-day mortality-associated independent factor and CVC removal as a protective factor. Garazi et al. [8] reported septic shock, carbapenem use (within the last 30 days), admission to the ICU and neutropenia as independent risk factors for mortality in their study of 102 patients. Araoaka et al. [11] demonstrated that independent risk factors for mortality were neutropenia and polymicrobial bacteraemia in their study of *Enterococcus spp.* in 53 patients. Hotta et al. [6] found that a higher SOFA score was a 30th-day mortality-associated independent risk factor in their analyses. Sumida et al. [7] found inadequate therapy and a SOFA score over six was associated with 30th-day mortality in univariate analyses in a group consisting of 30 patients, and they also detected the same factors as independent risk factors. Osawa et al. reported that admission to the ICU and mechanical ventilation support were risk factors for mortality [25].

We detected that the risk factors indicating the clinical severity or immunosuppression of patients during and before the diagnosis such as steroid use, advanced age, solid organ malignancy, Charlson’s morbidity score, SOFA, higher PBS and septic shock affected the 14th and 30th-day mortality rates. This finding supports the suggestion that the clinical severity of patients at diagnosis is as important as adequate treatment in terms of mortality [10-12].

Removal of current CVC was detected as an independent protective factor in our study. Similarly, several other studies found that CVC removal was a protective factor for mortality both in patients with CRB and other patients [10, 26].

A Pittsburgh bacteraemia score of ≥4 was found to be a prognostic risk factor for mortality in a previous study [27]. Likewise, we found that a PBS ≥4 was a prognostic risk factor for mortality.

We did not detect any difference in patient mortality between regimens including TMP/SMX and those including levofloxacin. Similarly, several other studies also found no difference in mortality between the two treatment regimens [23,28,29].

We found that TMP/SMX and levofloxacin combination did not significantly improve the prognosis in the present study. A previous study that assessed both in vitro and clinical outcomes showed that the combination is not superior to monotherapy [30]. 

## Conclusions

In conclusion, SM bloodstream infections result in higher mortality in patients with underlying disease and prognostic risk factors. Rates of adequate empirical treatment may be lower due to antibiotic resistance patterns. *Stenotrophomonas maltophilia *should be considered as the cause of infection in patients who have received long-term carbapenem treatment, those with solid organ or hematological malignancy, or SM colonization. In patients with SM bacteraemia, CVC should be removed immediately and antimicrobial therapy such as TMP/SMX or fluoroquinolone should be initiated.
